# A β2-Integrin/MRTF-A/SRF Pathway Regulates Dendritic Cell Gene Expression, Adhesion, and Traction Force Generation

**DOI:** 10.3389/fimmu.2019.01138

**Published:** 2019-05-28

**Authors:** Carla Guenther, Imrul Faisal, Liisa M. Uotila, Marc Llort Asens, Heidi Harjunpää, Terhi Savinko, Tiina Öhman, Sean Yao, Markus Moser, Stephan W. Morris, Sari Tojkander, Susanna Carola Fagerholm

**Affiliations:** ^1^Fagerholm Lab, MIBS, University of Helsinki, Helsinki, Finland; ^2^Institute of Biotechnology, University of Helsinki, Helsinki, Finland; ^3^Department of Veterinary Biosciences, University of Helsinki, Helsinki, Finland; ^4^Department of Molecular Medicine, Max Planck Institute of Biochemistry, Martinsried, Germany; ^5^Department of Pathology, St. Jude Children's Research Hospital, Memphis, TN, United States; ^6^Department of Hematology-Oncology, St. Jude Children's Research Hospital, Memphis, TN, United States

**Keywords:** dendritic cells, adhesion, MRTF-A, SRF, MKL-1, LAD-III, traction force

## Abstract

β2-integrins are essential for immune system function because they mediate immune cell adhesion and signaling. Consequently, a loss of β_2_-integrin expression or function causes the immunodeficiency disorders, Leukocyte Adhesion Deficiency (LAD) type I and III. LAD-III is caused by mutations in an important integrin regulator, kindlin-3, but exactly how kindlin-3 regulates leukocyte adhesion has remained incompletely understood. Here we demonstrate that mutation of the kindlin-3 binding site in the β2-integrin (TTT/AAA-β2-integrin knock-in mouse/KI) abolishes activation of the actin-regulated myocardin related transcription factor A/serum response factor (MRTF-A/SRF) signaling pathway in dendritic cells and MRTF-A/SRF-dependent gene expression. We show that Ras homolog gene family, member A (RhoA) activation and filamentous-actin (F-actin) polymerization is abolished in murine TTT/AAA-β2-integrin KI dendritic cells, which leads to a failure of MRTF-A to localize to the cell nucleus to coactivate genes together with SRF. In addition, we show that dendritic cell gene expression, adhesion and integrin-mediated traction forces on ligand coated surfaces is dependent on the MRTF-A/SRF signaling pathway. The participation of β2-integrin and kindlin-3-mediated cell adhesion in the regulation of the ubiquitous MRTF-A/SRF signaling pathway in immune cells may help explain the role of β2-integrin and kindlin-3 in integrin-mediated gene regulation and immune system function.

## Introduction

Leukocyte adhesion is an essential process in immunity. Adhesion is necessary for leukocyte surveillance in tissues, phagocytosis, lymphocyte homing, leukocyte extravasation, T-cell priming, and cytotoxic killing. One of the most important group of adhesion molecules on leukocytes are the β2-integrins. β2-integrins are transmembrane proteins consisting of the β-chain CD18 which forms heterodimers with the four α-chains: CD11a, CD11b, CD11c, and CD11d, forming 4 different receptors e.g., CD11a/CD18 (LFA-1, α_L_β_2_, ITAL Ag), CD11b/CD18 (Mac-1, α_M_β_2_, CR3, ITAM Ag), CD11c/CD18 (p150p95, α_X_β_2_, CR4, ITAX Ag), CD11d/CD18 (α_D_β_2_, ITAD Ag) (1).

β2-integrins participate in the leukocyte adhesion cascade where they mediate slow rolling and firm adhesion to the endothelium (2). During this process β2-integrins bind intercellular adhesion molecules (ICAMs) expressed on endothelial cells. In addition to ICAMs, β2-integrins also bind to a vast variety of other ligands such as complement protein iC3b during phagocytosis (3).

Integrins have been found to transmit signals bidirectionally, which means that they mediate inside-out and outside-in signaling during cell adhesion. On resting leukocytes integrins are typically in an inactive, bent conformation which prevents ligand binding (2). Intracellular signaling initiated for example by chemokine receptors eventually leads to integrin conformational changes into an active conformation that has an increased affinity for ligands (inside-out signaling) (4). Subsequent integrin-ligand binding results in further recruitment of integrins and cytoskeletal proteins to the adhesion site, followed by integrin-mediated outside-in signaling leading to further cellular changes.

Integrin conformational changes are regulated by binding of cytoplasmic cofactors to the cytoplasmic tail of the β2-integrin (5). The best characterized integrin cofactor is talin, which is associated with the active (high affinity) integrin conformation and mediates further binding of cytoplasmic proteins such as actin and focal adhesion kinase (6). Talin has been shown to mediate integrin downstream signaling under higher forces. Low and intermediate force transmission is mediated by another cytoplasmic integrin cofactor, kindlin (6).

The importance of adhesion molecules, their ligands and their cytoplasmic interaction partners is demonstrated in a group of immunodeficiencies termed leukocyte adhesion deficiencies (LAD). While LAD-I is caused by reduced or abolished expression of β2-integrins (7), LAD-III is caused by the mutation or absence of kindlin-3. LAD patients experience recurrent bacterial and fungal infections as well as severe bleeding (in LAD-III only) as a result of impaired leukocyte and platelet adhesion.

Kindlin-3-null mice have a similar phenotype as LAD-III patients, demonstrating the importance of kindlin-3 in integrin regulation (8). While it has been demonstrated that only a low level of kindlin-3 is required to enable proper β2-integrin function (9), the exact mechanism by which kindlin-3 regulates adhesion remains incompletely characterized (10). We have previously established a mouse model with a TTT/AAA β2-integrin knock-in mutation, which results in kindlin-3 being unable to bind to β2-integrins and consequently a loss of leukocyte adhesion (11). Furthermore, the reduced β2-integrin/kindlin-3 interaction leads to a range of phenotypic changes in leukocytes (11–13). Interestingly, TTT/AAA-β2-integrin KI dendritic cells (DCs) display an increased maturation phenotype. They express higher level of costimulatory markers such as CD40, CD80, CD86, chemokine receptors (CCR7) and produce more cytokines (IL-12, IL-10) than wild type cells. KI dendritic cells also display increased migration to lymph nodes and induce increased Th1 responses *in vivo* compared to WT dendritic cells (12). While these experiments indicate that active β2-integrins suppress the mature, migratory dendritic cell phenotype, the signaling pathways downstream of β2-integrins that mediate this phenotypic switch have not been identified.

SRF has been termed the master regulator of the cytoskeleton as this transcription factor regulates the expression of many cytoskeletal genes. The majority of SRF-mediated transcription of cytoskeletal genes has been shown to be dependent on its cofactor MRTF-A. In leukocytes, MRTF-A/SRF have been shown to regulate the expression of cytoskeletal proteins as well as β2-integrins (14–16). The MRTF-A/SRF pathway is activated in response to external cell stimuli which initiates F-actin polymerization downstream of RhoA activation. MRTF-A constantly shuttles between the cytoplasm and the nucleus but has been shown to be mainly cytoplasmic in resting cells. In the cytoplasm MRTF-A is bound to G-actin, thus upon F-actin polymerization MRTF-A is released and free to shuttle into the nucleus. Nuclear MRTF-A then initiates gene transcription together with SRF (17).

Here we show that kindlin-3-regulated β2-integrin adhesion is required for signaling via RhoA and actin to initiate MRTF-A nuclear localization in dendritic cells. Furthermore, dendritic cell adhesion, traction force generation and gene expression is regulated by MRTF-A/SRF signaling. These results may help explain the role of β2-integrins and kindlin-3 in gene regulation in leukocytes, leukocyte adhesion processes and immune responses.

## Methods

### Mice

Bone marrow was collected from euthanized male and female C57Bi/6NCrl (Charles River), previously described TTT/AAA β2-integrin knock-in mice (11) (8–39 weeks) and full MRTF-A knockout and control mice previously described in Cheng et al. (18). Fetal liver cells were collected from Kindlin-3^−/−^ and control mice. Experiments were performed according to Finnish Act on Animal Experimentation (62/2006) and approved by the Finnish National Animal Experiment Board. Kindlin-3^−/−^ and control mice were handled in strict accordance with regulations in Germany regarding the use of laboratory animals.

### Dendritic Cell Culture

Dendritic cells were generated by culturing bone marrow for 9–10 days (media change on day 3; 6 and 8) in 10 ng/ml GM-CSF (Peprotech) RPMI +10% FCS, 100 U/ml Pen/Strep and 2 mM L-glutamine. In some experiments, 10 μM CCG1423 (Cayman) was used to inhibit MRTF-A for 2 days before experiments.

### Immunohistochemistry

1x10^6^ dendritic cells on uncoated, iC3b (6 μg/ml; Calbiochem) or fibronectin (10 μg/ml; Calbiochem) coated coverslips were serum starved for 1 h with 0.3% FCS/RPMI, followed by serum stimulation (15% FCS 30 min). In adhesion stimulation experiment WT and KI dendritic cells were detached, serum starved in suspension for 1h and stimulated with replating the cells on glass coverslips or on iC3b coated coverslips for 1h. Cells were fixed with 4% PFA. F-actin content of 25–100 cells/animal was assessed via measurement of corrected total cell fluorescence (CTCF) of TRITC-phalloidin (Sigma) as described in Abashidze et al. (19). All slides were imaged using a Leica SP5 II (Leica Microsystems) LAS AF Lite Software, with 561 Laser (10% laser power). Z-stacks were taken with the following parameters: Spectral Range: 570–779 nm, QD405/488/561/635 mirror, Smart Gain 800 V, Smart Offset 0,0%, Pinhole 111.49 μm, Zoom: 1,00; Objective 63X, z-Distance 8.003μm, 55 steps, Format 512 × 512.

MRTF-A staining was performed on non-starved, serum starved, and serum stimulated cells. After fixation cells were permeabilized with 0,2% Triton-x/PBS for 5 min and stained for 1 h with anti MRTF-A (C-19, sc-21558, Santa Cruz Biotechnology) 1:100 in 1%FBS/PBS.

### RhoA Bound GTP Level

The RhoA activity was assessed via RhoA G-LISA Activation Assay Kit (Cytoskeleton Inc, Denver) according to the manufacturer's instructions. Samples were collected from serum starved cells to acquire a non-stimulated baseline level of GTP-bound RhoA (determined for each biological repeat and set to 1). Based on this value the fold change of RhoA activation was calculated for serum-stimulated cells (serum starvation followed by 15% FCS for 10 min). All samples were collected on ice and snap frozen in liquid nitrogen within 10 min after lysing the cells and stored at −80°C until G-LISA was run.

### Static Adhesion Assay

Static adhesion assay with resting and activated (200 nM PdBu, Sigma-Aldrich) cells at 0,2 x 10^6^ cells/ml on ICAM-1 (6 μg/ml; R&D Systems) and iC3b (6 μg/ml; Calbiochem) was performed as previously described (20). Rescue experiments with MRTF-A were performed after cell transfection as follows: CD11b, CD18 WT, CD18 TTT/AAA, MRTF-A^***^(21) in pCDNA3.1 vector or a combination of them were transfected into COS-1 cells using Xfect (Takara) following the manufacturer's guidelines. After transfection, the cells were incubated for 48 h at 37°C before static adhesion and western blot experiments.

### Western Blot

Transfected cells were lysed in M-PER lysis buffer (Thermo Scientific) in the presence of phosphatase and protease inhibitors (Pierce), and lysates were analyzed by Western blotting. Primary antibodies against MRTF-A, and against myc-tag were from Santa Cruz.

### Flow Cytometry

Following Fc block (eBiosciences, 93) cells were stained with the following antibodies: CD11a-PE (BioLegend, 2D7), CD18-FITC (BD Biosciences, C71/16) CD11c-PE-Cy7 (eBioscience, N418), CD11b-APC (BioLegend, M1/70), CD80-APC (eBioscience, 16-10A1), CCR7-PE (BioLegend, 4B12), CD40-PE (BioLegend, 3/23), CD86-FITC (BD Biosciences, GL1). FITC-conjugated antibodies were used at 1:100 dilution, all other antibodies were used at 1:200 dilution. Data were acquired using a LSRForetessa (BD) and analyzed using FlowJo software (TreeStar, USA).

### Traction Force Microscopy

200.000 dendritic cells in 2 ml whole media were incubated for 3h on iC3b (6μg/ml in PBS Calbiochem) -coated silicone-based gel substrates (Young's Modulus = 1 kPa)(Matrigen, USA).

For MRTF-A^−/−^ and control experiments, cells were plated on elastic polyacrylamide (PAA) gel substrates (Young's Modulus = 1 kPa) (22). Substrates were covered with sulfate fluorescent microspheres (Invitrogen, diameter 200 nm; excitation wavelength 488) before coating with iC3b. Sulfo-SANPAH (Sigma-Aldrich) was used as a linker in between the substrate and the coating. Cells were incubated on substrates for 1h prior to imaging. Single cells together with the underlying beads were imaged with 3I Marianas imaging system (3I intelligent Imaging Innovations, Germany) at +37°C in 5% CO_2_. A 63x/1.2 W C-Apochromat Corr WD = 0.28 M27 objective was used. Following live cell imaging, cells were detached from the gel substrates with 10 × Trypsin (Lonza) and a second set of nanobead images, serving as reference images, were obtained. Spatial maps of cell-exerted nanobead displacements were achieved by comparing the reference bead images together with the experimental images. With the knowledge of the bead displacement fields, substrate stiffness (1 kPa), and a manual trace of the cell boundary, the cell-exerted traction fields were computed by using Fourier Transform Traction Cytometry (23, 24). The root mean squared magnitude was computed from the traction field.

### Cell Migration Assays

3D migration assays were performed with μ-Slide Chemotaxis 3D (Ibidi) imaging slides according the manufacturer's protocol. Briefly, dendritic cells were mixed into a 1.5 mg/ml bovine collagen I (cellsystemsSlides) mix and injected into the slide's thin imaging strip. After 45min collagen polymerization, one of two chambers flanking the imaging strip were filled with media, and the other with media containing 1.25 μg/ml mCCL19 (R&D systems). One slide was used for each condition, assays were repeated 3 times. Dendritic cells were imaged using 3I Marianas imaging system (3I intelligent Imaging Innovations, Germany) by utilizing multipoint imaging. Controls were always imaged before treated or knock-in/-out dendritic cells. A 10x/0.30 EC Plan-Neofluar Ph1 WD = 5.2 M27 objective was used, the dish was placed in a heated sample chamber (+37°C), controlled for CO_2_. Cells were imaged using bright field, for 4 h every minute.

### qPCR

Total RNA was isolated from WT and WT MRTF-A inhibited dendritic cells with Nucleospin RNA kit (Macherey-Nagel) and converted into cDNA with High-Capacity cDNA Reverse Transcription kit (Thermo Fisher Scientific) according to the manufacturers' protocols. The quantitative Real-Time PCR (qRT-PCR) was performed by using Taqman chemistry. Briefly, the cDNA was amplified in a 11 μl volume containing 1 x TaqMan Fast Advanced Master Mix and predeveloped TaqMan primers and probes (CCR7 Mm01301785_m1, CD11a Mm00801807_m1). 18S rRNA (Eukaryotic 18S rRNA Endogenous Control, Thermo Fisher Scientific) was used as a reference gene and no template control (NTC) was included in each assay. Reactions were run with CFX96 Touch Real-Time PCR Detection System (Bio-Rad) with a program consisting of initial steps of 50°C for 2 min and 95°C for 20 s followed by 40 cycles of 95°C for 3 s and 60°C for 30 s. Each sample was run in triplicate. qRT-PCR data was analyzed by using CFX Maestro (Bio-Rad) and finalized with Excel(Microsoft). Relative units were calculated by using the comparative C_T_ method which has been described elsewhere (25).

### mRNA Sequencing

The sequencing was performed at the Biomedicum Functional Genomics Unit (FuGU) of the University of Helsinki with the Illumina NextSeq500 using a NextSeq Mid Output 150 cycle flow cell. From 2μg of total RNA from each biological replicate (4 MRTF-A^−/−^ and 4 MRTF-A^+/+^, age matched), Library preparation was done using a NEBNext® Ultra™ II Directional RNA Library Prep Kit for Illumina (E7760) and mRNA enrichment was done by NEBNext® Poly(A) mRNA Magnetic Isolation Module (E7490), according to manufacturer's protocols. 10 PCR cycles were used. Libraries' concentrations were determined by Qubit, while quality and size distribution were analyzed using Bioanalyzer 2100. Fastq files are deposited at NCBI's Gene Expression Omnibus (GEO) database with BioProject ID PRJNA535475 and BioSample accession number SAMN11506732.

### RNA-Seq Data Analysis

The sequencing quality of raw RNA-Seq reads from fastq files was evaluated by FastQC (http://www.bioinformatics.babraham.ac.uk/projects/fastqc/). Sequencing reads were then aligned against the mouse reference genome (*Mus musculus* GRCm38.95) by HISAT2 (26). Transcripts were counted using HTSeq (27). Differential expression analysis was performed by the R Bioconductor package DESeq2 (28) using <0.05 as cutoff for the adjusted *p*-value. Heatmaps were generated from normalized expression (regularized log transformation) values for individual samples that were obtained from DESeq2.

### Pathway Enrichment Analysis

All the differentially expressed genes having adjusted *p*-value < 0.05 (calculated by DESeq2) were ranked by their log2 fold-changes. Pathway enrichment analysis for GO biological process of the ranked gene list was done with the GSEA software version 3.0 (29) against C5 gene ontology biological process dataset (version 6.2) downloaded from molecular signature database (MSigDB). The GSEA output was further processed and visualized using EnrichmentMap (version 3.2.0) on Cytoscape (version 3.7.1). In addition, pathway enrichment analysis for reactome pathways was performed by g:Profiler (30).

### Statistics

The Student's 2-tailed *t*-test and Mann-Whitney test, were used to calculate statistical significance in prism. For traction force microscopy and migration assays Mann-Whitney tests were used. All *p*-values are shown as < 0.05^*^, < 0.01^**^, < 0.005^***^.

## Results

### RhoA Activation and F-Actin Polymerization Following Extracellular Stimuli Are Reduced in TTT/AAA-β2-Integrin KI Dendritic Cells

β2-integrin-mediated leukocyte adhesion to integrin ligands requires the interaction between β2-integrin and kindlin-3 (11, 12). Reduced β2-integrin/kindlin-3 interaction in TTT/AAA-β2-integrin KI dendritic cells leads to marked changes in gene expression and cellular reprogramming to a migratory phenotype (12). To identify pathways downstream of kindlin-3 that are involved in integrin/kindlin-3-regulated gene expression in dendritic cells, we performed Ingenuity Pathway Analysis (IPA) of gene expression data from WT and KI dendritic cells (12). This software combines algorithms with published datasets of primary literature, public and third-party databases to identify molecular interactions, causations and model pathways. As indicated in Table 1, the MRTF-A/SRF pathway was predicted to be inhibited as a consequence of the reduced β2-integrin/kindlin-3 interaction in TTT/AAA-β2-integrin KI dendritic cells.

**Table 1:** IPA was performed with gene expression profiles from KI, *N* = 3.

**Upstream regulator**	**Molecule type**	**Predicted activation state**	***p*-value of overlap**
SRF	Transcription regulator		3,01E-10
MRTF-B/MKL2	Transcription regulator	Inhibited	1,88E-09
MRTF-A/MKL1	Transcription regulator	Inhibited	4,24E-09

**Figure 1:**
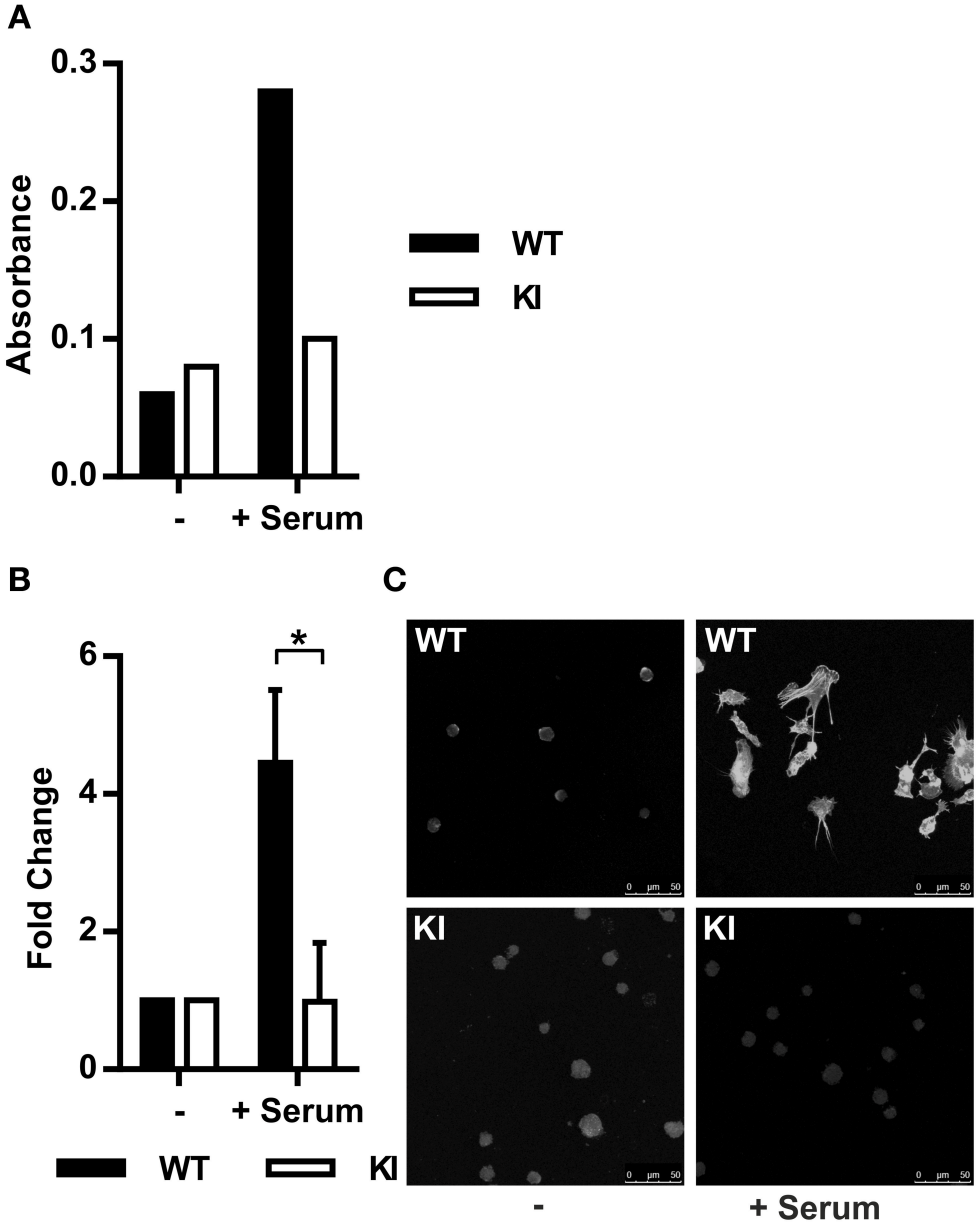
Abolished β2-integrin/kindlin-3 link leads to impaired actin dynamics and impaired nuclear MRTF-A shuttling. **(A)** RhoA activity in WT and KI dendritic cells was measured in serum starved cells (0.3% fetal calf serum (FCS)/RPMI for 1 h) and serum-stimulated cells (15% FCS for 10 min). *N* = 3, one representative result is shown. **(B)** F-actin content was assessed via measurement of corrected total cell fluorescence (CTCF) of TRITC-phalloidin stained cells. Baseline level of F-actin was acquired from serum starved cells and F-actin fold change was calculated for 15% FCS-stimulated cells (30 min stimulation). 25–100 cells per condition were measured, *N* = 3. **(C)** Immunofluorescence images of serum starved and serum-stimulated WT and KI cells stained with TRITC-phalloidin. *p < 0.05.

The MRTF-A/SRF pathway is regulated by actin dynamics in cells. G-actin-bound MRTF-A remains cytoplasmic, whilst RhoA-induced F-actin polymerization releases this transcription factor. MRTF-A is subsequently free to translocate into the nucleus where it initiates transcription of cytoskeletal genes together with SRF (17, 31, 32). Based on the IPA prediction that the MRTF-A/SRF pathway is inhibited in KI dendritic cells, we first set out to investigate RhoA activity and actin polymerization in WT and KI cells. Indeed, RhoA activity in serum-stimulated cells was lower in KI cells compared to WT cells (Figure 1A) and in addition, serum-stimulated F-actin polymerization was significantly decreased in KI dendritic cells compared to WT dendritic cells (Figures 1B,C). These results show that β2-integrins are upstream of RhoA-mediated F-actin polymerization in dendritic cells.

### MRTF-A Nuclear Shuttling Is Impaired as a Result of Mutated CD18 or Deleted Kindlin-3

**Figure 2:**
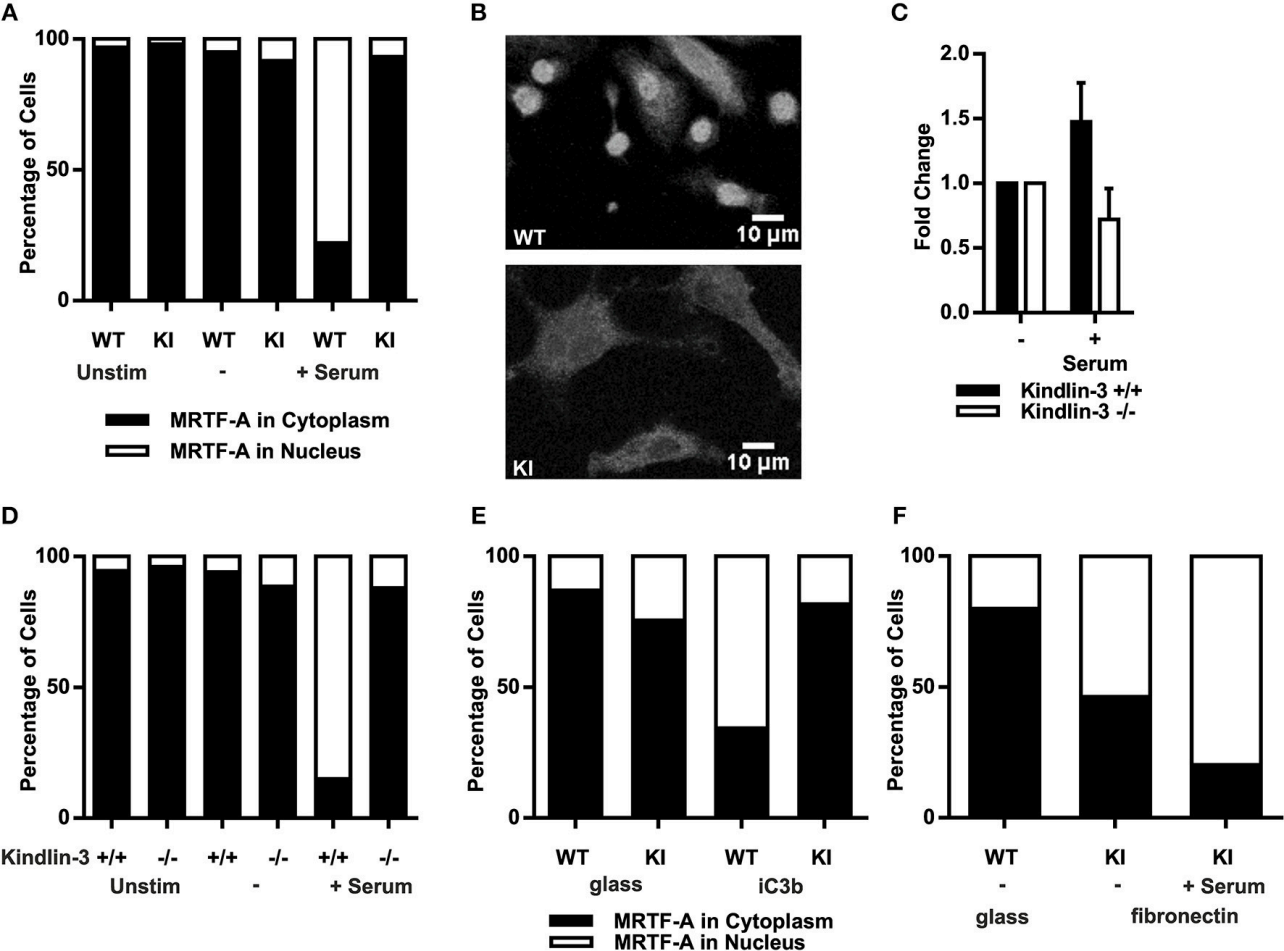
Abolished β2-integrin mediated adhesion leads to impaired nuclear MRTF-A shuttling. **(A)** Total percentages of dendritic cells with nuclear MRTF-A and cytoplasmic MRTF-A are shown. *N* = 4 and 200 cells per condition were analyzed. MRTF-A staining was performed on non-starved, starved and serum stimulated cells and **(B)** immunofluorescence images of WT and KI cells are shown after serum stimulation. **(C)** F-actin content as CTCF of WT and kindlin-3^−/−^ 25–100 dendritic cells per condition were measured, *N* = 2. **(D)** Total percentages of kindlin-3^−/−^ and WT dendritic cells with nuclear MRTF-A and cytoplasmic MRTF-A are shown. **(E)** Total percentages of adhesion on iC3b induced nuclear MRTF-A and cytoplasmic MRTF-A are shown. WT and KI dendritic cells were detached, serum starved in suspension, and stimulated with replating on glass coverslips or on iC3b coated coverslips. **(F)** Total percentages of WT dendritic cells starved on glass compared to KI dendritic cells seeded overnight on fibronectin with nuclear MRTF-A and cytoplasmic MRTF-A. KI dendritic cells were serum starved and stimulated. **(A–F)** If not otherwise indicated: *N* = 3 and 200 cells per condition were analyzed. MRTF-A staining was performed on non-starved, starved, and serum stimulated cells.

As F-actin polymerization initiates MRTF-A nuclear shuttling, allowing regulation of gene transcription together with SRF (33, 34) we investigated serum-induced MRTF-A shuttling in dendritic cells. In resting as well as serum starved dendritic cells MRTF-A was mainly cytoplasmic independent of functional β2-integrin/kindlin-3 interaction, as expected. Serum stimulation led to MRTF-A translocation into the nuclei of WT dendritic cells, however in KI dendritic cells MRTF-A remained predominantly cytoplasmic (Figures 2A,B).

These results confirm the IPA results and suggest that RhoA activity, actin dynamics and MRTF-A shuttling are dependent on functional β2-integrins in dendritic cells. However, these results do not exclude the possibility that except for kindlin-3, also other cytoplasmic factors that bind to the TTT site in the β2-integrin cytoplasmic tail may regulate this pathway. Therefore, in order to further pinpoint the β2-integrin cofactor involved in activating the MRTF-A/SRF pathway we analyzed F-actin polymerization and MRTF-A shuttling in Kindlin-3 ^−/−^ dendritic cells. Indeed, Kindlin-3^−/−^ dendritic cells showed impaired F-actin polymerization and MRTF-A shuttling similar to KI cells (Figures 2C,D).

To examine whether integrin/kindlin complexes *per se*, or integrin- and kindlin-3-regulated adhesion, is responsible for regulating MRTF-A nuclear shuttling, we investigated whether the β2-integrin ligand iC3b could induce MRTF-A nuclear shuttling in DCs. Indeed, placing DCs on iC3b allowed for MRTF-A nuclear shuttling in WT but not β2-integrin KI DCs (Figure 2E). However, placing the β2-integrin KI cells on fibronectin-coated surfaces, which allows for β1-integrin-mediated adhesion of these cells, allowed for MRTF-A nuclear shuttling both in the absence and presence of serum (Figure 2F). These results show that integrin-mediated adhesion, rather than integrin/kindlin signaling, is responsible for the shuttling of this transcription factor in DCs.

These results confirm that MRTF-A nuclear shuttling is regulated by kindlin-3 regulated, β2-integrin-mediated cell adhesion, which in turn mediates RhoA activation, F-actin polymerization and MRTF-A nuclear shuttling in dendritic cells. These results further confirm the bioinformatics data showing that β2-integrin/kindlin-3 regulates MRTF-A/SRF-mediated gene expression in dendritic cells.

### Inhibition of MRTF-A in WT Dendritic Cells Does Not Regulate Dendritic Cell Costimulatory Molecule Expression or Cell Migration in 3D

**Figure 3:**
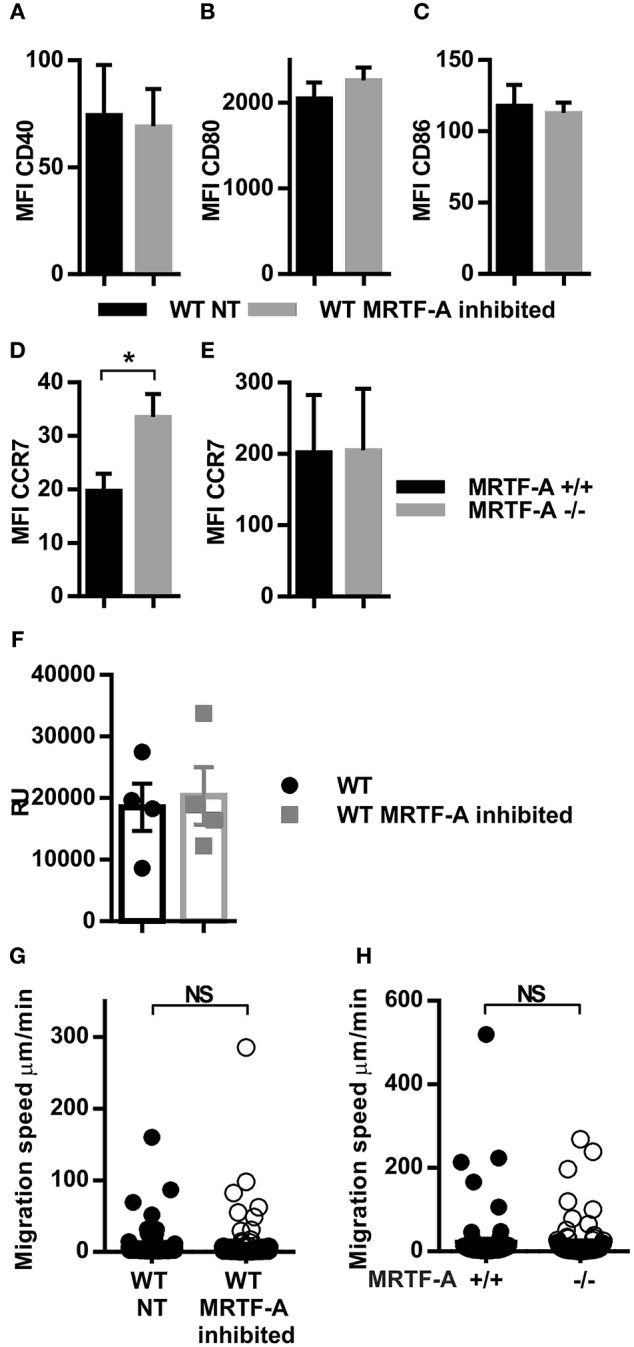
Inhibition but not deletion of MRTF-A leads to altered CCR7 expression and no change in migration speed. **(A)** CD40 **(B)** CD80 **(C)** CD86 and **(D)** CRR7 expression in WT NT and MRTF-A inhibited dendritic cells (*N* = 6); **(E)** CCR7 expression of MRTF-A ^−^/^−^ and MRTF-A^+/+^ (*N* = 5); surface marker expression was determined by flow cytometry. **(F)** CCR7 mRNA level of MRTF-A inhibited and NT WT dendritic cells determined by qPCR (*N* = 4). **(G)** Scatter plots of tracked WT NT and WT MRTF-A inhibited and **(H)** MRTF-A^−^/^−^ and MRTF-A^+/+^ dendritic cell speeds in a 3D collagen matrix toward CCL19 are shown. Only cells faster than 1 μm/min have been evaluated as migratory and used for the analysis. *p < 0.05.

We have previously shown that the β2-integrins negatively regulate dendritic cell maturation, which is associated with increased surface expression of CD40, CD80 and CD86 costimulatory molecules and the chemokine receptor CCR7 in TTT/AAA-β2-integrin KI cells (12). CCR7 is the receptor for chemokines CCL19 and CCL21 which guides dendritic cells to the lymph nodes where they engage in T-cell priming. In order to investigate whether the MRTF-A/SRF pathway is involved in regulating dendritic cell maturation downstream of β2-integrins, we inhibited MRTF-A with a specific inhibitor, CCG1423 in WT dendritic cells and analyzed phenotypic changes by flow cytometry (Figure 3). We did not find significant changes in CD40, CD80, or CD86 expression in CCG1423-treated WT dendritic cells as compared to WT non-treated dendritic cells. MRTF-A inhibition, led to a significant increase in CCR7 expression on the cell surface in DCs, although mRNA levels were not affected (Figures 3D–F). CCR7 levels were not increased in MRTF-A ^−/−^ DCs (Figure 3E).

MRTFA/SRF has been previously linked to cell migration in neutrophils, lymphoblasts and hematopoetic stem cells (14, 35, 36). We therefore asked the question whether the MRTF-A/SRF pathway regulates dendritic cell migration. We compared dendritic cell migration speeds in a 3D collagen matrix after MRTF-A inhibition or deletion in the presence of chemokine CCL19 (Figures 3G,H). Surprisingly, we did not find a significant difference between migration speed either after MRTF-A inhibition with CCG1423 nor as a consequence of MRTF-A deletion (utilizing MRTF-A^−/−^) in dendritic cells (Figures 3G,H). In summary we have found that the MRTF-A/SRF pathway in dendritic cells is downstream of β2-integrins but does not regulate dendritic cell expression of costimulatory molecules, nor cell migration in 3D.

### MRTF-A Regulates Gene Expression of Cell Cycle, Lipid Metabolism, and Cytoskeletal Genes in Dendritic Cells

**Figure 4:**
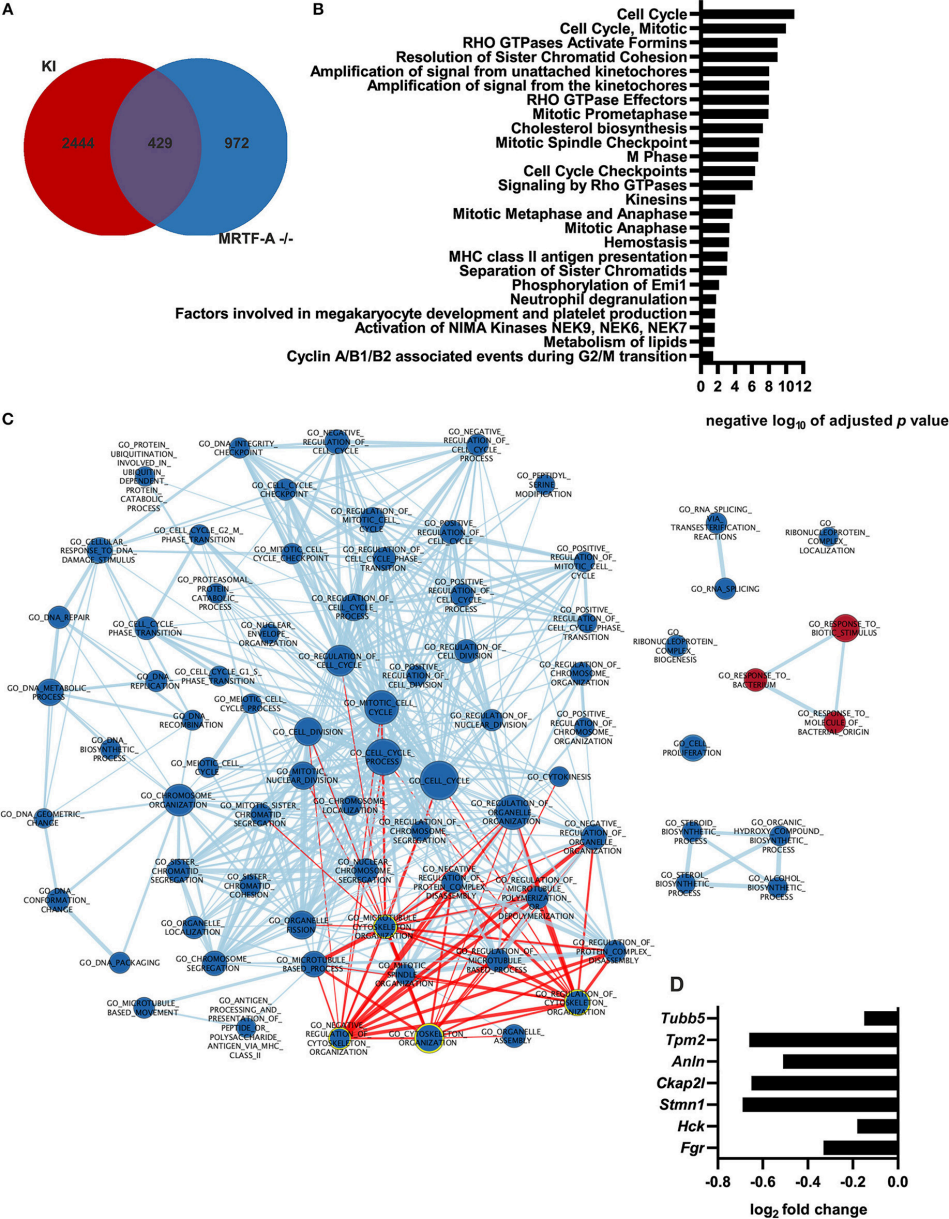
Gene expression profile of MRTF-A^−/−^ cells and comparison with integrin KI. **(A)** Depicted is the overlap of differently regulated genes shared by the MRTF-A^−^/^−^ vs. ^+/+^ and the KI vs. WT RNA-Seq data. **(B)** Ranked list of negative logarithm of adjusted *p*-values from the reactome pathway enrichment analysis. Pathway was performed using g:Profiler. **(C)** Node map of pathway enrichment analysis for GO biological process of the GSEA ranked gene list. Analysis is based on RNA-Seq data derived from MRTF-A^−^/^−^ and ^+/+^ (*N* = 4). Nodes highlighted in red are associated with the cytoskeleton. Nodes highlighted with yellow outline contain word “cytoskeleton.” **(D)** Depiction of cytoskeletal genes that are differently regulated in KI and MRTF-A^−^/^−^.

Although the MRTF-A/SRF pathway is often described as the “master regulator of the cytoskeleton,” the role of the MRTF-A/SRF pathway in regulation of gene expression in dendritic cells has not previously been investigated. To generate more in-depth understanding of the role of the MRTF-A/SRF pathway in dendritic cells, we therefore performed RNA-Seq analysis of MRTF-A^−/−^ cells (Supplementary Table 1, Supplementary Figure 1). 1401 genes were found to be differentially expressed between WT and MRTF-A^−/−^ cells. 429 of the MRTF-A-regulated, differentially expressed genes are also regulated by β2-integrins in DCs (12) (Figure 4A). The main pathways affected were cell cycle-related, but also for example lipid metabolism genes appear to be regulated by MRTF-A. However, also cytoskeletal genes and Rho GTPase signaling were significantly affected by MRTF-A deletion in dendritic cells (Figures 4B,C). Several cytoskeletal genes and cytoskeletal modulators, such as Fgr, Hck, Stmn1, Ckap2l, Anln, Tpm2, Tubb5, are downregulated both in MRTF-A^−/−^ and TTT/AAA-β2-integrin KI dendritic cells (Figure 4D). Together, these data further indicate that β2-integrin and MRTF-A are on a common pathway regulating gene expression in dendritic cells.

### MRTF-A Inhibition Results in Decreased Dendritic Cell Adhesion

**Figure 5:**
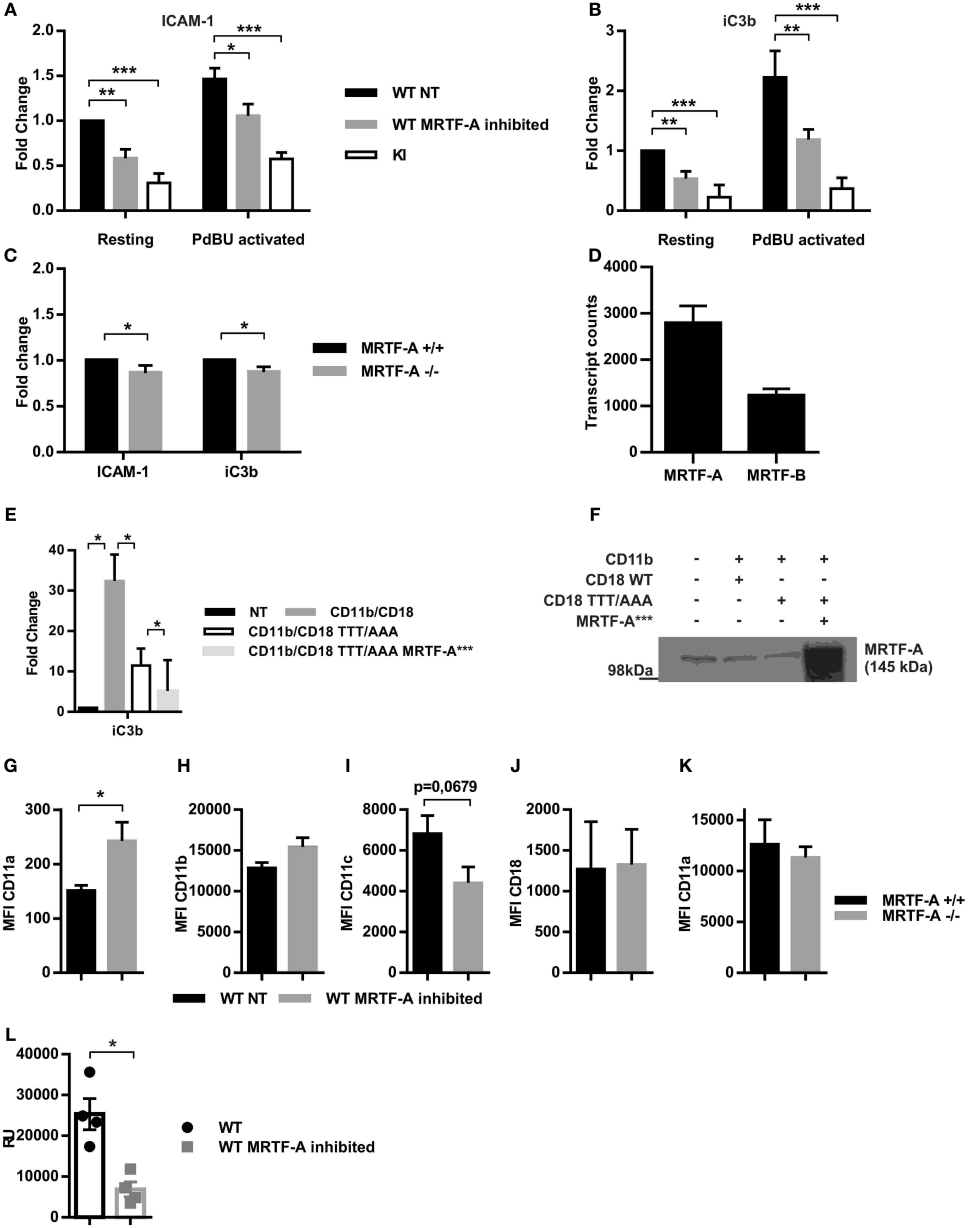
Inhibition of the MRTF-A/SRF pathway leads to reduced adhesion independent of β2-integrin expression. The effect of MRTF-A inhibition on dendritic cell adhesion to **(A)** ICAM-1 and **(B)** to iC3b was compared to adhesion of TTT/AAA-β2-integrin KI cells. Adhesion of resting, non-treated (NT) WT dendritic cells was set to 1 and fold changes for all other conditions was calculated, *N* = 5. **(C)** MRTF-A ^−^/^−^ dendritic cell adhesion to ICAM-1 and iC3b was compared to adhesion of MRTF-A^+/+^ littermates, *N* = 4. **(D)** mRNA level of MRTF-A and MRTF-B in MRTF-A^+/+^ mice (*N* = 4) based on RNA-Seq data. **(E)** TTT/AAA rescue experiment with constitutively active MRTF-A (MRTF-A^***^). Adhesion of non-treated COS-1 cells was set to 1 and fold change for adhesion of COS-1 cells transfected with CD11b/CD18, CD11b/CD18-TTT/AAA (CD18 featuring the TTT/AAA mutation), and CD11b/CD18-TTT/AAA plus MRTF-A^***^. **(F)** Depiction of conditions used in rescue experiment. **(G)** CD11a, **(H)** CD11b, **(I)** CD11c and **(J)** CD18 expression in WT NT and MRTF-A inhibited dendritic cells (*N* = 6). **(K)** CD11a expression in MRTF-A^−^/^−^ and^+/+^ dendritic cells (*N* = 5). **(L)** CD11a mRNA level of MRTF-A inhibited and NT WT dendritic cells determined by qPCR (*N* = 4). *p < 0.05, **p < 0.01.

One of the most prominent phenotypical changes of TTT/AAA-β2-integrin KI dendritic cells is their reduced adhesion. Many cytoskeletal proteins are necessary for cell adhesion. We hypothesized that the reduced adhesion in KI cells may be partly due to the suppression of the MRTF-A/SRF pathway and subsequent changes in expression of cytoskeletal genes. We thus analyzed static adhesion of MRTF-A inhibited dendritic cells to β2-integrins ligands ICAM-1 and iC3b. As shown in Figures 5A,B, β2-integrin-mediated adhesion of MRTF-A inhibited WT dendritic cells was significantly reduced, although not as drastically as in TTT/AAA-β2-integrin KI cells. A similar reduction in adhesion was seen in MRTF-A^−/−^ dendritic cells (Figure 5C). There are two MRTF isoforms present in most cells: MRTF-A and MRTF-B (33). RNA-Seq analysis confirmed that murine dendritic cells express both MRTF-A and MRTF-B, with MRTF-A being expressed at higher levels than MRTF-B (Figure 5D). The difference in adhesion of the MRTF-A ^−/−^ dendritic cells as compared to inhibitor-treated cells could thus be explained by MRTF-B partly compensating for MRTF-A functions in these cells.

To investigate whether MRTF-A-regulated gene expression by itself could rescue adhesion in TTT/AAA-β2-integrin KI cells, we cotransfected COS-cells with TTT/AAA-β2-integrins and a constitutively active form of MRTF-A that can enter the nucleus without cell stimulation (21) and investigated cell adhesion in these cells. MRTF-A-^***^ did not rescue TTT/AAA-β2-integrin-mediated adhesion in transfected COS cells, indicating that this pathway alone is not sufficient to drive cell adhesion when kindlin cannot bind to the integrin to induce integrin activation (Figures 5E,F). Nevertheless, these results show that the MRTF-A/SRF pathways contributes to β2-integrin-mediated dendritic cell adhesion to ligands.

β2-integrins themselves have been shown to be negatively regulated by SRF and MRTF-A in that lack of either transcription factor results in CD11b overexpression in neutrophils (14, 36). In hematopoetic stem cells SRF deletion results in integrin overexpression (35). We thus set out to clarify if the reduced adhesive phenotype following MRTF-A inhibition was due to downregulation of β2-integrins. We measured surface expression levels of the β2-integrins β2-chain (CD18) and three of the α-chains, CD11a, CD11b, and CD11c (Figures 5G–K). Surprisingly we found that none of these β2-integrin subunits was significantly decreased and CD11a was even significantly increased following MRTF-A inhibition (Figure 5G), although qPCR showed reduced levels of expression of CD11a (Figure 5L). MRTF-A deletion did not affect CD11a surface expression levels (Figure 5K). Therefore, reduction of β2-integrin mediated adhesion following MRTF-A inhibition is not due to reduced β2-integrin expression.

### The β2-Integrin/MRTFA/SRF Pathway Contributes to Integrin-Mediated Traction Force Generation

**Figure 6:**
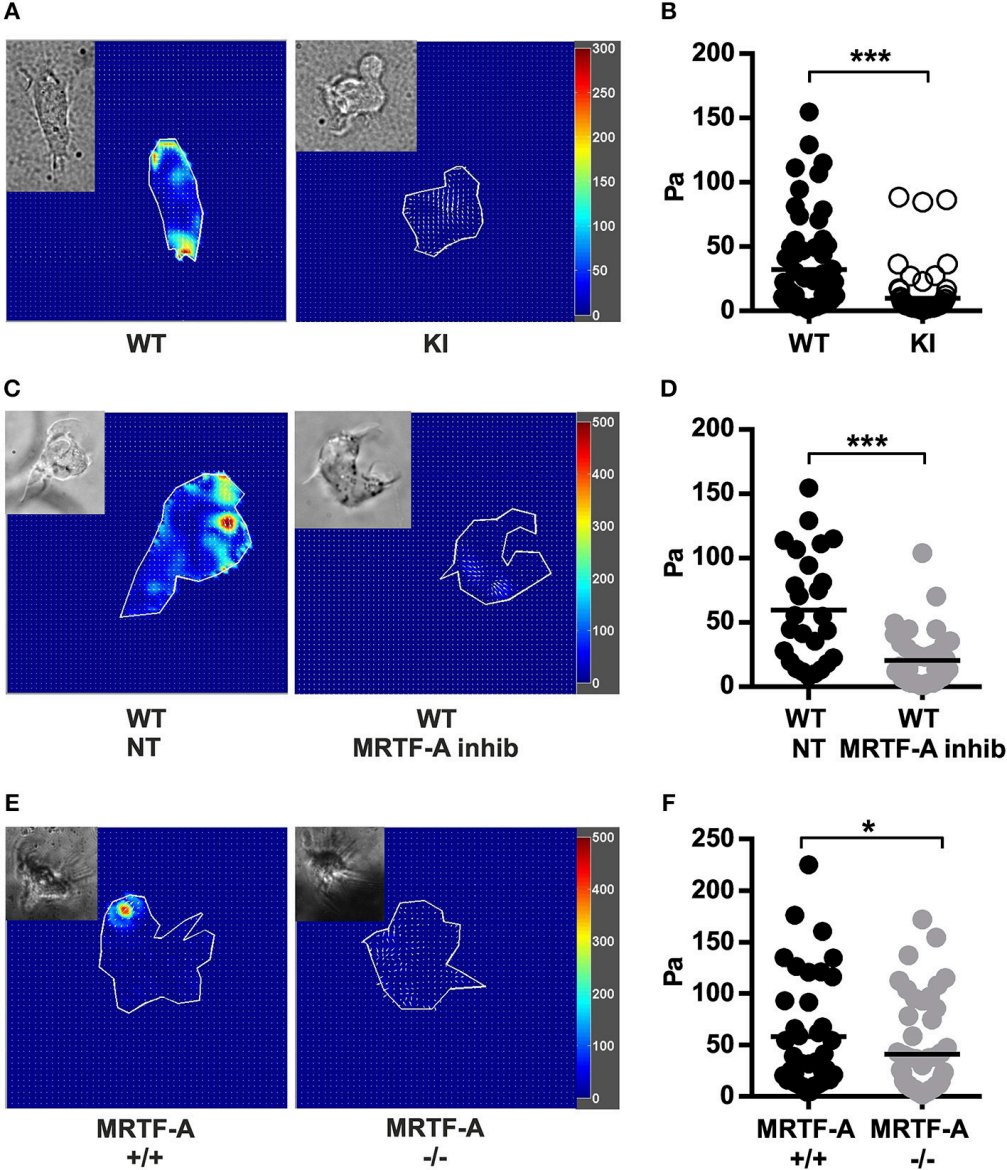
Traction force generation in dendritic cells is regulated by the β2-integrin/MRTF-A/SRF pathway. **(A)** Heatmaps depicting traction force generated by WT and TTT/AAA KI dendritic cells and **(C)** non treated WT and MRTF-A inhibited WT dendritic cells. **(B)** Scatter plots of analyzed WT and KI dendritic cells and **(D)** non treated WT and MRTF-A inhibited WT dendritic cell traction forces (Pa) are shown. Three experiments were performed, 25 cells were measured each time and a total of 75 cells analyzed. **(E)** Heatmaps depicting traction force generated by an example MRTF-A^+^/^+^ and ^−/−^ dendritic cells. **(F)** Scatter plots of analyzed MRTF-A^+^/^+^ and ^−/−^ dendritic cells *N* = 4. *p < 0.05, ***p < 0.005.

Integrins function as bidirectional transmitters of mechanical forces across cell membranes. To investigate whether the β2-integrin/kindlin-3 interaction regulates force transmission through the integrin, we performed traction force microscopy experiments. By comparing the beads' positions on top of which cells were bound to iC3b to the same bead structure without the cell we could measure how much the cell deforms the bead-containing hydrogel. This deformation is due to traction forces generated during adhesion and the force of the traction force can be calculated from bead deformation and hydrogel substrate stiffness. Traction force microscopy experiments comparing TTT/AAA-β2-integrin KI to WT dendritic cells revealed that KI dendritic cells generate significantly reduced traction forces on β2-integrin ligand coated substrates compared to WT dendritic cells (Figures 6A,B).

In order to investigate whether traction force generation was dependent on the MRTF-A/SRF pathway downstream of β2-integrin/kindlin-3, we performed traction force microscopy experiments on MRTF-A inhibited WT dendritic cells. Interestingly, MRTF-A inhibition as well as deletion resulted in a similar decrease of integrin-mediated traction forces on ligand coated surfaces as disruption of the β2-integrin/kindlin-3 interaction (Figures 6C–F). Together, these results show that the β2-integrin/kindlin-3/MRTF-A/SRF pathway regulates integrin-mediated cell adhesion as well as integrin-generated cellular traction forces in dendritic cells.

In summary we have found that the MRTF-A/SRF pathway in dendritic cells is downstream of β2-integrin/kindlin-3 interactions and that this pathway regulates dendritic cell gene expression, cell adhesion and traction force generation, but not dendritic cell migration in 3D.

## Discussion

Although β2-integrins are abundantly expressed in dendritic cells, the role of β2-integrins in dendritic cell functions is relatively poorly understood. Dendritic cell migration from tissues to lymph nodes, which occurs in a 3D environment, can occur without integrins (37). In addition, β2-integrins have been shown to suppress the mature, migratory dendritic cell phenotype (12). However, the signaling pathways downstream of β2-integrins that mediate this phenotypic switch have not been identified.

In this study we have identified the β2-integrin as an upstream regulator of the MRTF-A/SRF pathway. Our results show that β2-integrin-mediated, kindlin-3-regulated cell adhesion leads to RhoA activation to regulate F-actin polymerization and MRTF-A nuclear shuttling. This allows MRTF-A to coactivate genes together with SRF. Although MRTF-A has previously been implicated as a main player in regulating cytoskeletal genes in other cell types, we show here by RNA-Seq analysis that MRTF-A also regulates gene expression related to cell cycle, lipid metabolism, and many other pathways in dendritic cells. In macrophages, MRTF-A/SRF mediate cytoskeletal gene expression (14, 36, 38), whilst in B cells SRF deletion led to decreased level of IgM, CD19, and CXCR4 expression (39). Thus our results confirm the involvement of MRTF-A/SRF in the regulation of gene expression in immune cells, however the effect on gene expression appears highly cell-type specific.

We show that MRTF-A inhibition or deletion significantly decreases adhesion to β2-integrin ligands ICAM-1 and iC3b but does not impact on dendritic cell 3D migration speed in response to chemokine stimulation. MRTF-A and SRF have been shown to activate gene expression of proteins associated with focal adhesions in fibroblasts and are associated with migration and invasion in a cancer cell line (16). In hematopoietic stem cells SRF regulates adhesion and integrin expression (35). In neutrophils, SRF has been shown to be essential for adhesion and extravasation (36), whilst in macrophages SRF independent gene targets of MRTF-A were predicted to be focal adhesion associated (40). Deletion of MRTF-A in neutrophils leads to impaired 2D migration associated with altered adhesion (14). Therefore, our results together with published studies confirm that MRTFA/SRF transcription factors play an important role in regulating the adhesive phenotype of myeloid immune cells.

We have linked the reduced adhesion to reduced traction forces upon MRTF-A inhibition. This result could be interpreted as that the integrin/kindlin-3 interaction is necessary for overall generation of traction forces, because the integrin/kindlin interaction is required for optimal integrin function. Traction forces are generated by interaction of the (actin-) cytoskeleton with myosin. Both actin and myosin are MRTF-A/SRF target genes. We thus hypothesize that reduced adhesion following MRTF-A inhibition in dendritic cells may be due to altered cytoskeletal organization which ultimately renders the dendritic cells less able to generate integrin-mediated traction forces. An alternative explanation would be that kindlin-3 and talin interaction with the β2-integrin is regulated by SRF, as was shown in SRF^−/−^ neutrophils (36). This alternative would extend the regulatory feedback loop between β2-integrin/kindlin-3, F-actin and MRTF-A back to the integrin regulators kindlin-3 and talin.

Interestingly, like β2-integrin deficiency and kindlin-3 deficiency, which lead to LAD-syndromes in man, MRTF-A deficiency in man has recently been shown to lead to immunodeficiency with prominent susceptibility to bacterial infections (14). Patient neutrophil and lymphocyte functions, such as migration, was impaired, and patient dendritic cells displayed impaired spreading and podosome formation (14), as we have previously reported in TTT/AAA-β2-integrin KI dendritic cells (41). The similarities of symptoms in human MRTF-A deficiency and β2-integrin deficiency, together with the results presented here, indicate that some of the functions of integrins and kindlin-3 in immune cells, which are impaired in LAD patients may involve regulation of transcription through the MRTF-A/SRF pathway. We hypothesize that abnormal expression of actin cytoskeleton genes due to specific defects in the MRTF-A/SRF pathway in LAD cells may contribute to immune defects (e.g., defects in myeloid cell adhesion, migration, phagocytosis, etc) in LAD diseases.

MRTF-A has previously been shown to regulate the development of specific adipocyte subpopulations (42) and is essential for thymocyte development (39). SRF deletion in hematopoetic stem cells led to accumulation of CD11b positive cells and thus an increase in macrophage like cells (35). Interestingly, cell adhesion, which maintains active MRTF-A/SRF signaling, has recently been shown to be essential to prevent progenitor cells from switching to the adipocyte lineage during cardiomyocyte differentiation (43). Mechanistical data illustrating the role of SRF and MRTF-A in the regulation of cellular identity has been gathered from neural and liver cells. In these cells, induced MRTF-A nuclear accumulation resulted in downregulation of cell-type specific genes, changes in epigenetic status of regulator elements as well as chromatin organization (44). It is interesting to note that in dendritic cells, our gene expression profiling data now show that MRTF-A regulates genes that are associated with the cell cycle and with lipid metabolism, indicating new roles of this pathway in metabolism and cell proliferation/differentiation in immune cells, topics for further studies in the future.

In summary, our results indicate that the β2-integrin-regulated MRTF-A/SRF pathway is a key regulatory element in dendritic cells that regulates gene expression associated with cell cycle, lipid metabolism and the cytoskeleton. β2-integrins regulate MRTF-A/SRF signaling and expression of cytoskeletal genes, and enables the adhesive dendritic cell phenotype and integrin-mediated traction force generation. We thus propose that the β2-integrin-regulated MRTF-A/SRF pathway may function as a roadblock that needs to be overcome in dendritic cells for them to initiate the phenotypic switch from an adhesive to a mature, migratory phenotype.

## Ethics Statement

Experiments were performed according to Finnish Act on Animal Experimentation (62/2006) and approved by the Finnish National Animal Experiment Board. Kindlin-3^−/−^ and control mice were handled in strict accordance with regulations in Germany regarding the use of laboratory animals.

## Author Contributions

SF planned the study. CG performed the experiments with assistance of LU and TS. MA performed MRTF-A rescue experiments. HH performed qPCR. ST supervised the traction force microscopy experiments. MM provided kindlin-3^−/−^ and +/+ fetal liver cells. SM provided MRTF-A −/− and +/+ mice. CG, ST, and SY analyzed experimental data. TÖ performed ingenuity pathway analysis of WT and KI and IF analyzed MRTF-A^−/−^ and +/+ RNA-Seq data. CG wrote the paper together with SF.

### Conflict of Interest Statement

The authors declare that the research was conducted in the absence of any commercial or financial relationships that could be construed as a potential conflict of interest.

## Supplementary Material

Supplementary Table 1 List of differentially expressed genes from the RNA-Seq data.

Supplementary Figure 1 Heatmap of gene expression changes.
