# Supplementary figures and images for: A β2-Integrin/MRTF-A/SRF Pathway Regulates Dendritic Cell Gene Expression, Adhesion, and Traction Force Generation
Source: Front Immunol. 2019 May 28;10:1138. doi: 10.3389/fimmu.2019.01138 (PMC6546827; doi:10.3389/fimmu.2019.01138)

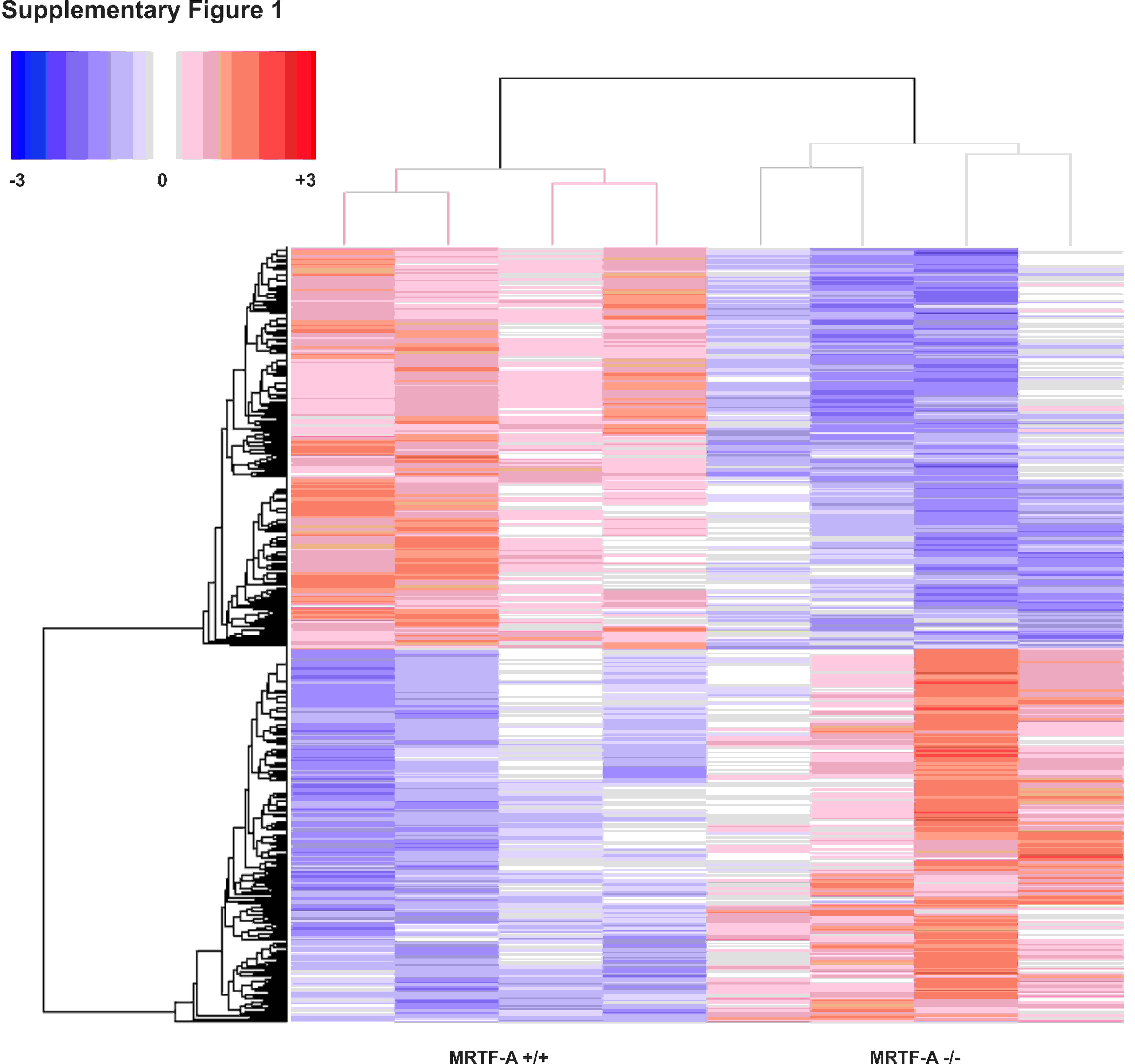

Supplement: Supplementary Figure 1 — Heatmap of gene expression changes. [file Image_1.jpg]
